# The Most Deadly Disease

**Published:** 1875-03

**Authors:** 


### THE MOST DEADLY DISEASE.

The most deadly acute disease from which the people of the United States are to-day suffering, is pneumonia. There are not less than 2,000 cases at this hour, in the city of New York alone. Many are nearly down with it who do not suspect it, and these can bring it on by a single act of indiscretion. Ten minutes on a street corner in the cold wind; a glass of brandy or whisky; late hours and exposure at night; an evening in a badly ventilated church or theatre; any of these may permit the latent disease to manifest itself.

The disease attacks the lungs, but is not, as generally supposed, a species of hasty consumption. There is very little expectoration in pneumonia, and in many cases none at all. The cold settles on the lungs, the air-passages fill up with mucus, and death is due to the impossibility of breathing, or to the weakness which the disease brings on, as cautious dieting is necessary. When the trouble in the lungs is overcome, the patient is often left in so low a condition that it is impossible to make him rally. It is a rather singular phase of this deadly disease that the percentage of cases is as four to one in favor of men. Women very seldom suffer from it. This may be due to the greater exposure to which men are subjected, and to the more sedentary life of women, who do not suffer from such constant changes and such shocks to the lungs. The best preventive against pneumonia is to keep the mouth closed when going from a hot place to a cold, and breathe through the nose. It comes like a flash of lightning; there is no preparation or means of averting it. One may go to bed healthy, to all appearances, and wake up with the disease in full blast. Then it is simply a question of constitution. Medical skill avails but little, and physicians pursue but one course—to keep the patient in a warm, equal temperature; to give remedies as much as possible to clear the lungs, and to seek to keep up the proper animal heat. The patient ordinarily partly loses consciousness on the third day, and the crisis is reached on the seventh. If not dead then, there is a small chance of recovery, and all depends on the strength of the patient.

Pneumonia is far more fatal with us than it was years ago. We may attribute the increased mortality from this disease to a multitude of causes. Alcohol gives the disease more victims than all else. Other causes are steam-heating devices, bad ventilation, and tobacco-smoke. The devitalized heat of the steam-pipes is most injurious to the lungs. The action of the heat on the iron coils sends off a deleterious gas, which seriously impairs the lungs and renders the inhalation of cold air positively dangerous. Tobacco-smoke dries up the mucous membrane of the throat and air-passages and dispels their healthy action. Alcohol destroys the power of the stomach, and so lessens vitality that a simple "cold" speedily becomes pneumonia. These causes—added to the absurd custom of bundling up the throat while leaving the feet nearly without protection—are sufficient to account for the enormous mortality from this disease.

---

From bad to worse is poor improvement.

